# Ginsenosides Act As Positive Modulators of P2X4 Receptors[Fn FN5]

**DOI:** 10.1124/mol.118.113696

**Published:** 2019-02

**Authors:** Kshitija Dhuna, Matthew Felgate, Stefan M. Bidula, Samuel Walpole, Lucka Bibic, Brett A. Cromer, Jesus Angulo, Julie Sanderson, Martin J. Stebbing, Leanne Stokes

**Affiliations:** School of Health and Biomedical Sciences, RMIT University, Bundoora, Victoria, Australia (K.D., B.A.C., M.J.S., L.S.); School of Pharmacy, University of East Anglia, Norwich Research Park, Norwich, United Kingdom (M.F., S.M.B., S.W., L.B., J.A., J.S., L.S.); Department of Chemistry and Biotechnology, Swinburne University of Technology, Hawthorn, Victoria, Australia (B.A.C.); and Florey Institute of Neuroscience and Mental Health, Department of Anatomy and Neuroscience, University of Melbourne, Victoria, Australia (M.J.S.)

## Abstract

We investigated the selectivity of protopanaxadiol ginsenosides from *Panax ginseng* acting as positive allosteric modulators on P2X receptors. ATP-induced responses were measured in stable cell lines overexpressing human P2X4 using a YOPRO-1 dye uptake assay, intracellular calcium measurements, and whole-cell patch-clamp recordings. Ginsenosides CK and Rd were demonstrated to enhance ATP responses at P2X4 by ∼twofold, similar to potentiation by the known positive modulator ivermectin. Investigations into the role of P2X4 in mediating a cytotoxic effect showed that only P2X7 expression in HEK-293 cells induces cell death in response to high concentrations of ATP, and that ginsenosides can enhance this process. Generation of a P2X7-deficient clone of BV-2 microglial cells using CRISPR/Cas9 gene editing enabled an investigation of endogenous P2X4 in a microglial cell line. Compared with parental BV-2 cells, P2X7-deficient BV-2 cells showed minor potentiation of ATP responses by ginsenosides, and insensitivity to ATP^−^ or ATP^+^ ginsenoside-induced cell death, indicating a primary role for P2X7 receptors in both of these effects. Computational docking to a homology model of human P2X4, based on the open state of zfP2X4, yielded evidence of a putative ginsenoside binding site in P2X4 in the central vestibule region of the large ectodomain.

## Introduction

P2X receptors are a family of ATP-gated nonselective cation channels of which there are seven known subunits (P2X1–7) with varying expression patterns (North, 2002). Their physiological roles range from the regulation of membrane potential and intracellular calcium concentration (all P2X receptors) to the regulation of mediator secretion such as interleukin 1*β* (IL-1*β*) and brain-derived neurotrophic factor (BDNF) by P2X7 and P2X4, respectively. Investigations into drugs that can modulate the activity of P2X receptors have been quite intensive in the last decade, with successful identification of selective antagonists for many of the P2X family, as reviewed in Bartlett et al. (2014) and Stokes et al. (2017). We recently reported the identification of a series of positive allosteric modulators of P2X7 found in extracts of the traditional Chinese medicinal plant, *Panax ginseng* (Helliwell et al., 2015). In this work, we have further investigated the selectivity of ginsenosides for P2X7 within the P2X family, focusing on purinergic receptors typically coexpressed with P2X7 in immune cells, namely P2X4, P2Y1, and P2Y2 (Bowler et al., 2003).

P2X4 is one of the most ubiquitously expressed P2X receptors (Soto et al., 1996) and has been implicated in several physiological pathways in different tissues. Prominent expression of P2X4 has been demonstrated in endothelial cells, immune cells, and neurons, as reviewed in Stokes et al. (2017). An important role for P2X4 in vasodilation responses to shear stress was elucidated in 2000 (Yamamoto et al., 2000), and transgenic mice lacking P2X4 later confirmed a role in nitric oxide production and vessel remodelling (Yamamoto et al., 2006). In the central nervous system (CNS), P2X4 has been implicated in long-term potentiation (Sim et al., 2006) and in the pathophysiology associated with neuropathic pain (Tsuda et al., 2003; Coull et al., 2005). P2X4 expressed on spinal cord microglia is involved in activation of microglia and release of mediators, including BDNF, which alter sensory neuronal pain transmission pathways (Coull et al., 2005; Ulmann et al., 2008). Also in the CNS, a role for P2X4 has been described in alcohol-intake behavior due to regulation of the dopamine reward pathway in the brain (Asatryan et al., 2011; Franklin et al., 2015; Khoja et al., 2016). Finally, in the immune system, P2X4 plays a role in the regulation of CXCL5 production and secretion from monocytes and macrophages (Layhadi et al., 2018).

Many of the roles for P2X4 have been elucidated using transgenic P2X4^−/−^ mice or short hairpin RNA knockdown of the receptor because selective and potent antagonists for P2X4 have only recently been described. These include PSB-12062, BX430, NP-1815-PX, and 5-(3-Bromophenyl)-1,3-dihydro-2H-benzofuro[3,2-e]-1,4-diazepin-2-one (5-BDBD) (Hernandez-Olmos et al., 2012; Balázs et al., 2013; Ase et al., 2015; Matsumura et al., 2016; Stokes et al., 2017). In contrast to antagonists, relatively few positive allosteric modulators (PAMs) have been described for P2X receptors. Possibly the best known PAM for P2X receptors is ivermectin, which has most activity at P2X4 (Khakh et al., 1999b; Priel and Silberberg, 2004), although it also has some reported positive modulator activity on human P2X7 (Nörenberg et al., 2012). Other than ivermectin, cibacron blue, tenidap, clemastine, progesterone, and tetrahydrodeoxycorticosterone have been identified as positive modulators for P2X4, P2X7, and P2X2, respectively (Miller et al., 1998; Sanz et al., 1998; De Roo et al., 2010; Nörenberg et al., 2011). In addition, trace metals such as zinc and copper have PAM activity at several P2X receptors, including P2X2 and P2X4, as reviewed by Coddou et al. (2011a,b).

Ginsenosides are triterpenoid saponins found in the root extract of plants belonging to *P. ginseng*. Two subclasses of ginsenosides exist, designated protopanaxadiols (PPD) and protopanaxtriols (PPT) due to the positioning of glycoside attachments on the molecules. Ginsenosides have been reported to act on a number of ion channels such as voltage-gated Na^+^ and K^+^ channels, GABA_A_, and 5-HT_3_ receptors (Nah, 2014). Other constituents of ginseng such as the glycolipoprotein gintonin have been shown to potentiate P2X1 receptors (Choi et al., 2013). In our previous study (Helliwell et al., 2015), we demonstrated that the major ginsenoside metabolite generated in vivo, compound K (CK), in addition to protopanaxadiol ginsenosides Rd, Rb1, and Rh2, acts on P2X7 to potentiate the action of ATP. Ginsenosides reduced the EC_50_ for the physiologic agonist, ATP, at mouse and human P2X7 and enhanced ATP-induced cell death in the J774 mouse macrophage cell line (Helliwell et al., 2015). In this study, we demonstrate that the protopanaxadiol ginsenosides Rd and CK have a similar potentiating action on P2X4, most likely through a highly similar binding site identified in the central vestibule region; however, the magnitude of the potentiating effect on P2X4 is less than at P2X7.

## Materials and Methods

### 

#### Materials.

ATP (A7699; Sigma-Aldrich, St. Louis, MO) was prepared in distilled water and adjusted to pH 7.4 with 5 M NaOH. Aliquots were stored at −20°C and used only once. 5-(3-Bromophenyl)-1,3-dihydro-2H-benzofuro[3,2-e]-1,4-diazepin-2-one (5-BDBD) (Tocris Biosciences, Bio-Techne Ltd., Abingdon, U.K.) was prepared in dimethylsulfoxide (DMSO) at 10 mM and stored at −20°C. PSB-12062 (SML0753; Sigma-Aldrich) was prepared in distilled water to 10 mM. Ivermectin (Tocris Biosciences) was prepared at 30 mM in DMSO and stored at −20°C. Ginsenosides CK, Rd, Rb1, Rh2, and protopanaxadiol (PPD) (99% purity) were purchased from Shanghai Richem International Ltd., Shanghai, China, and 10 mM stocks were prepared in sterile DMSO (Sigma-Aldrich) in glass vials. Stock solutions were stored at −20°C. The G115 standardized ginseng extract was a gift of Professor C. C. Xue (RMIT University, Australia) and was prepared in water, as described in Helliwell et al. (2015).

#### Cell Culture.

Stably transfected HEK-293 cells were maintained in Dulbecco’s modified Eagle’s medium (DMEM):F12 media containing L-glutamine (cat. no. 11320-034; Life Technologies, Fisher Scientific, Waltham, MA) supplemented with 10% fetal bovine serum (US origin, cat. no. F2442; Sigma-Aldrich) and 100 U/ml penicillin plus 100 *µ*g/ml streptomycin (Fisher Scientific, Waltham, MA). Stably transfected 1321N1 cells were maintained in DMEM high-glucose media supplemented with 10% fetal bovine serum and 100 U/ml penicillin plus 100 *µ*g/ml streptomycin. To maintain stable expression of P2X constructs, 400–800 *µ*g/ml geneticin (Gibco, Fisher Scientific) was added to the culture media. Initial transient transfections of HEK-293 cells were performed in 35-mm petri dishes using 0.1–1 *μ*g plasmid DNA (Glu-Glu-tagged hP2X4 or hP2X7 in pcDNA3) and Lipofectamine 2000 (3 *µ*l/*μ*g DNA) complexes in OptiMem media (Fisher Scientific). Cells were plated into 96-well plates (cat. no. 167008; Nunc, Fisher Scientific, Waltham, MA) precoated with 50 *µ*g/ml poly-D-lysine (Merck Millipore, Burlington, MA), and plates were incubated overnight before experiments.

Mouse microglial cell line BV-2 (a gift of Dr. B. Gu, Florey Institute of Neurosciences, Melbourne) was maintained in DMEM:F12 media containing L-glutamine (cat. no. 11320-034; Life Technologies) supplemented with 10% fetal bovine serum (Sigma-Aldrich US origin, F2442) and 100 U/ml penicillin plus 100 *µ*g/ml streptomycin (Life Technologies).

#### CRISPR/Cas9 Gene Editing.

Complementary RNA was designed against exon 2 of mouse *P2RX7*, and two RNA constructs were purchased from Dharmacon (GE Healthcare, Chicago, IL). BV-2 cells (120,000 cells/well) in a six-well plate were transfected with endotoxin-free 1 *µ*g EditR-Cas9 plasmid with blasticidin resistance (GE Healthcare Dharmacon, Lafayette, CO) plus 25 nM target RNA and 25 nM complementary RNA for *P2RX7* using Dharmafect DUO reagent (4 *µ*l). Following 24-hour incubation, media were replaced, and, after an additional 72-hour incubation, blasticidin was added to a final concentration of 1 *µ*g/ml. Antibiotic selection was performed for 5 days, and remaining cells were left to expand for analysis of gene editing. Cells were tested for lack of P2X7 expression using flow cytometry. Successful wells showing lack of P2X7 expression underwent single-cell cloning in 96-well plates, and multiple clones were screened for lack of expression. Genomic DNA was extracted from successful clones and amplified by polymerase chain reaction for the *P2RX7* exon 2 region. Polymerase chain reaction products were sent for sequencing to verify mutations in this region (Eurofins Genomics, Ebersberg, Germany).

#### Flow Cytometry and Immunofluorescence.

BV-2 cells (5 × 10^5^ cells) were stained with rat anti-mouse P2X7 antibody (Hano43; Enzo Life Sciences UK Ltd, Exeter, U.K.) at 1:10 dilution in cold phosphate-buffered saline (PBS)/0.5% bovine serum albumin (BSA) buffer. Staining was performed on ice for 1 hour. Cells were washed with PBS/0.5% BSA buffer and stained with a goat anti-rat IgG Alexa488 secondary antibody (Fisher Scientific) at 1:200 dilution for 1 hour on ice. Following washing with PBS/0.5% BSA buffer, cells were resuspended in 300 *µ*l PBS/0.5% BSA buffer for acquisition on a FACSCalibur or on a Cytoflex flow cytometer.

For immunofluorescence, cells were grown overnight on 13-mm glass coverslips and washed with PBS prior to fixation with 4% paraformaldehyde (Sigma-Aldrich). Cells were permeabilized using PBS/0.5% BSA buffer containing 0.1% saponin (Sigma-Aldrich) and blocked for 1 hour. Rabbit anti-P2X4 (Alomone Laboratories, Jerusalem, Israel) was used at 1:200 dilution in PBS/0.5% BSA buffer, and cells were stained overnight. Following washing, anti-rabbit IgG Alexa 568 was used at 1:200 dilution in PBS/0.5% BSA buffer. Coverslips were mounted onto glass slides using Prolong Gold antifade containing 4′,6′-diamidino-2-phenylindole (Fisher Scientific). A Zeiss AxioPlan 2ie upright fluorescent microscope was used to take the images.

#### Western Blotting.

Whole-cell lysates were prepared in radioimmunoprecipitation assay lysis buffer (Fisher Scientific) containing protease inhibitors (Fisher Scientific). Cell pellets were disrupted with a pipette tip and lysed on ice for 30 minutes. Lysates were then cleared by centrifugation at 10,000*g* for 10 minutes, and supernatants were transferred into clean tubes. Protein was measured using a bicinchoninic acid assay (Pierce, Fisher Scientific, Waltham, MA) with BSA as standard (2 mg/ml to 20 ng/ml). Absorbance at 562 nm was measured using a Flexstation 3 plate reader (Molecular Devices, San Jose, CA). A quantity amounting to 25 *µ*g total protein was loaded per lane onto a Bolt 4%–12% Bis–Tris gel (Fisher Scientific) using the supplied gel-loading buffer plus reducing agent. Electrophoresis was performed using 1× MES buffer. Protein was transferred onto polyvinylidene difluoride membrane (Immobilon P; Fisher Scientific) using semidry transfer for 60 minutes. Membranes were blocked with 5% nonfat milk solution in Tris-buffered saline/Tween 20 (TBST) overnight at 4°C. Primary antibodies were incubated for 2 hours at room temperature in 5% nonfat milk solution in TBST. Anti-P2X4 (APR-004; Alomone Laboratories) and anti-P2X7 C-terminal antibody (APR-002; Alomone Laboratories) were both used at 1:2000 dilution. A goat anti-rabbit IgG-horseradish peroxidase secondary (Sigma-Aldrich) was used at 1:2000 dilution. Blots were stripped, washed, reblocked, and then reprobed for *β*-actin using a mouse monoclonal anti–*β*-actin antibody (A5316; Sigma-Aldrich) at 1:2000 dilution in 5% nonfat milk solution in TBST. An anti-mouse IgG-horseradish peroxidase (Sigma-Aldrich) secondary was used at 1:10,000 dilution. Luminata chemiluminescent substrate (Merck Millipore) was used to develop blots, and images were taken using an Image Quant LAS 4000 imager.

#### Dye Uptake Experiments.

For YOPRO-1 dye uptake experiments, cells were plated at a density of 2 × 10^4^ cells/well in complete DMEM:F12 media (100 *µ*l per well) in poly-D-lysine–coated 96-well plates. Media were removed using a manual multichannel pipette and replaced with a low divalent cation buffer (145 mM NaCl, 2 mM KCl, 13 mM D-glucose, 10 mM HEPES, and 0.1 mM CaCl_2_, pH 7.3) containing 2 *µ*M YO-PRO-1 iodide (cat. no. Y3663; Life Technologies). For preincubation experiments, the cells were treated with ginsenosides (10 *µ*M) in YO-PRO–containing low divalent cation buffer. For the majority of experiments, ginsenosides were coinjected simultaneously with the agonist using a Flexstation 3 microplate reader (Molecular Devices). Ginsenosides and agonist were prepared at 10× final concentration in the compound plate. Dye uptake over time was recorded using an excitation wavelength of 488 nm and an emission wavelength of 520 nm on the Flexstation 3 (six reads/well, photo-multiplier tube (PMT) setting medium). Basal fluorescence measurements were acquired for 40 seconds, followed by automatic injection of agonist, and the kinetic measurement of fluorescence intensity was performed for 300 seconds using Softmax Pro v5.4 software. Dye uptake responses were calculated as area under the curve from 50 to 300 seconds using zero baseline normalized data.

#### Intracellular Calcium Measurements.

HEK-293, HEK-hP2X4 cells, and 1321N1-hP2X4 cells in poly-D-lysine–coated 96-well plates were loaded with 2 *µ*M Fura-2 acetoxymethyl ester (Fisher Scientific or HelloBio, Bristol, U.K.) in Hanks’ balanced salt solution buffer containing 250 *µ*M sulfinpyrazone (Sigma-Aldrich) for 40–60 minutes at 37°C. Following loading, buffer was removed using a multichannel pipette and replaced with standard extracellular buffer or low divalent buffer. Cells were warmed for 10 minutes before measurements were started. Fura-2 was measured at excitation wavelengths 340 and 380 nm with emission wavelength 520 nm using a Flexstation 3 plate reader. Sampling interval was 3.5 seconds and three reads/well. Fura-2 ratio was calculated using Softmax Pro v5.4, and responses were measured using area under curve kinetic reduction.

Calcium responses in parental and P2X7-deficient BV-2 cells were performed using a Fura-2-QBT kit (Molecular Devices). Reagent was prepared in low divalent buffer containing 250 *µ*M sulfinpyrazone and added to cells plated into poly-D-lysine–coated 96-well plates for 60 minutes (180 *µ*l/well). Using the Fura-2-QBT kit eliminated the washing step from the procedure, ensuring that no cells were lost from the plate. Fura-2 fluorescence was measured at excitation wavelengths 340 and 380 nm with emission wavelength 520 nm using a Flexstation 3 plate reader. Sampling interval was 3.5 seconds and three reads/well. Fura-2 ratio was calculated using Softmax Pro v5.4, and responses were measured using area under curve kinetic reduction. Conditions were applied in triplicate, and experiments were performed four independent times.

#### Cell Viability.

Viability experiments were performed using the CellTiter 96 Aqueous Non-Radioactive Cell Proliferation assay (G3580, Promega, Madison, WI), a colorimetric method for determining the number of viable cells. HEK-293 cells were plated at 5 × 10^4^ cells/well in complete DMEM:F12 media and left for 24 hours under normal growth conditions. Treatments were then added to the cells (2× final concentration) and incubated for an additional 24 hours. 3-(4,5-dimethylthiazol-2-yl)-5-(3-carboxymethoxyphenyl)-2-(4-sulfophenyl)-2H-tetrazolium, inner salt (MTS) solution (20 *µ*l) was added to media in each well 1–4 hours before the stipulated end point time. Absorbance was measured at 490 nm using a Clariostar plate reader (BMG Labtech Ltd., Aylesbury, U.K.) or a Flexstation 3 plate reader.

#### Patch Clamping.

HEK-293 cells stably expressing hP2X4 were plated onto 13-mm glass coverslips 24 hours before recording ATP-induced membrane currents in the whole-cell patch clamp configuration. An EPC10 amplifier (HEKA Elektronik, Harvard Bioscience Inc., Lambrecht/Pfalz, Germany) was used to voltage clamp cells at −60 mV. Borosilicate glass electrodes (World Precision Instruments, Hitchin, U.K.) had a resistance of 5–8 MΩ when filled with standard internal solution (145 mM NaCl, 10 mM HEPES, 10 mM EGTA, pH 7.3, with 5 M NaOH). Cells were continually perfused by a gravity feed with standard extracellular buffer solution (145 mM NaCl, 5 mM KCl, 2 mM CaCl_2_, 1 mM MgCl_2_, 13 mM glucose, 10 mM HEPES, pH 7.3) prior to seal formation. ATP and ginsenosides were applied using a computer-controlled fast-flow system (Bio-Logic Science Instruments, Seyssinet-Pariset, France) with the perfusion capillaries placed in close proximity to the cell under investigation. Cells were dialyzed with internal solution for 1 minute, and ATP in the absence or presence of ginsenoside was applied every minute for four applications.

#### Molecular Modeling and Docking.

The coordinates of zebrafish P2X4 in the ATP-bound open state (Hattori and Gouaux, 2012) (Protein Data Bank: 4DW1) were used as a template. The sequence of human P2X4 was aligned to the template using the ClustalW algorithm (Larkin et al., 2007). The Schrödinger Prime software (www.schrodinger.com) was used to construct an energy-based all-atom model with the OPLS3 force field keeping the ATP coordinates from the template. Three-dimensional models of the ginsenosides CK and Rd were generated using LigPrep software within the Schrödinger Maestro suite (www.schrodinger.com). The OPLS3 forcefield was used to generate 32 low-energy conformers for each ginsenoside.

Induced fit docking was performed using the automated extended sampling protocol in the Schrödinger Maestro suite (Sherman et al., 2006). This first performs several initial docking runs in which side chains are either trimmed, or their Van der Waals potentials softened according to their flexibility. Side chains are then rebuilt, and those within 5 Å of the ligand are optimized using Prime. Ligands were then redocked to the new receptor structure using the Glide SP algorithm (Friesner et al., 2004) and standard potentials. Structures within 30 kcal mol^−1^ of the lowest energy structure were retained. The receptor grid was centered on the highest-scoring potential binding site, found using Sitemap (Halgren, 2007), and had cubic box dimensions of 30 Å. Each of the 32 conformers was docked using the extended sampling protocol. For each ginsenoside, the resulting poses were clustered by heavy atom root-mean-square deviation (RMSD) using the average-linkage method, and a representative structure was chosen from the model closest to the centroid of the most populated cluster. For CK and Rd, the most populated cluster made up 46% and 82% of all solutions, respectively.

#### Statistical Analysis.

Graphs were plotted using GraphPad Prism versions 6 or 7 (La Jolla, CA). Concentration–response curves were fitted using a log (agonist) versus response–variable slope (four parameter) best-fit equation. Data were analyzed for statistical significance using one-way analysis of variance (ANOVA) or two-way ANOVA with post hoc tests as appropriate (GraphPad Prism). Significance was taken as *P* < 0.05.

## Results

**Fig. 1.**
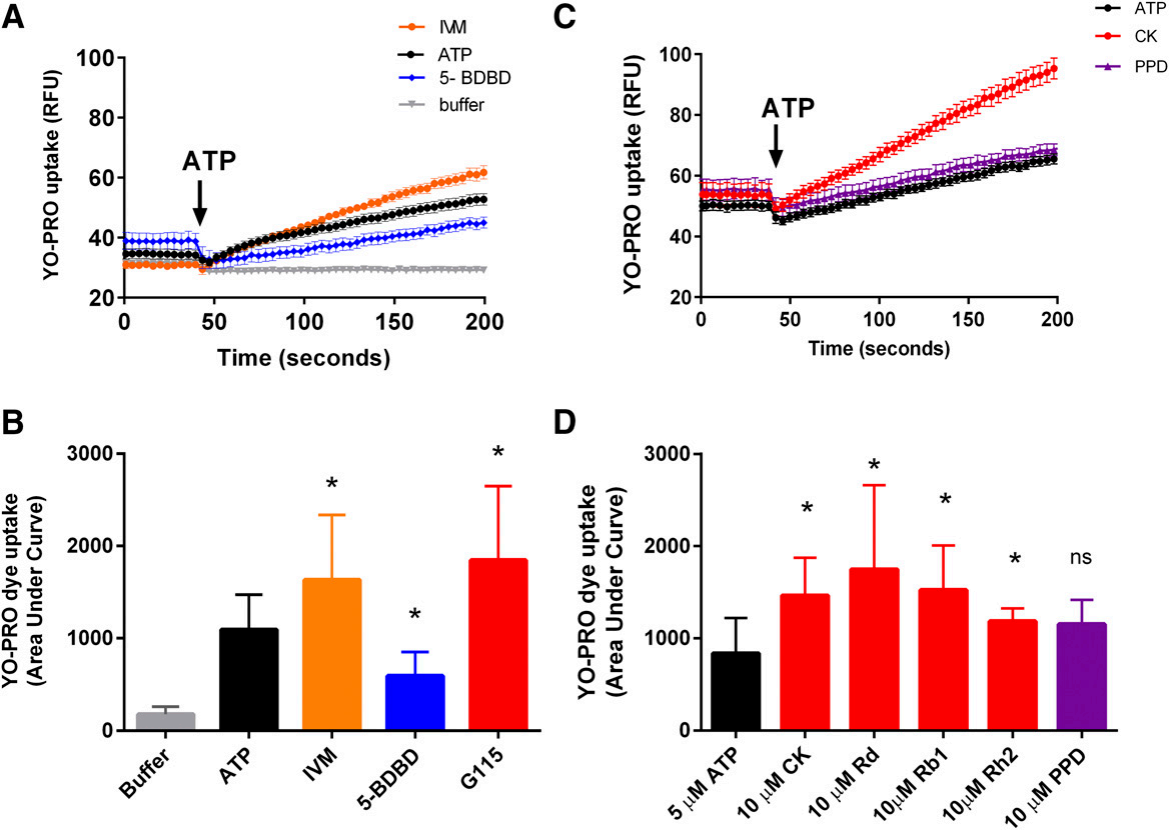
Protopanaxadiol ginsenosides and G115 increase hP2X4-mediated YOPRO-1 uptake responses. (A) Representative data showing YOPRO-1 uptake in response to ATP (5 *µ*M) in the presence of ivermectin (IVM, 3 *µ*M) or 5-BDBD (20 *µ*M). (B) Summary area under curve data from normalized YOPRO-1 uptake in HEK-hP2X4 cells (*n* = 3 independent experiments). Error bars represent S.D.; *represents *P* < 0.05 by one-way ANOVA with Dunnett’s multiple comparison post hoc test. (C) YOPRO-1 uptake responses in HEK-hP2X4 cells to ATP (5 *µ*M) in low divalent buffer solution (black) or ATP plus PPD (purple; 10 *µ*M) and ATP plus CK (red; 10 *µ*M). Ginsenosides were coinjected with the agonist ATP. (D) Data have been quantified as area under dye uptake curve (50–180 seconds). Bar graphs show data from three to five independent experiments. Error bars represent S.D.; *represents *P* < 0.05 by one-way ANOVA with Dunnett’s multiple comparison post hoc test, ns represents not significant.

For our investigation into the selectivity of protopanaxadiol ginsenosides for P2X7, we chose the closely related P2X4 receptor because this is known to be coexpressed with P2X7 in macrophages and other immune cells. We first generated a HEK-293 cell line stably expressing a Glu-Glu C-terminal tagged human P2X4 (HEK-hP2X4) and established an assay to measure P2X4-mediated secondary pore responses via YOPRO-1 dye uptake using a Flexstation 3 plate reader. The ATP-induced YOPRO-1 dye uptake response could be increased by ivermectin (3–5 *µ*M) and reduced by the known P2X4 antagonist 5-BDBD (20 *µ*M) (Fig. 1). This confirms that P2X4 receptors can elicit a large pore response in HEK-293 cells (Bernier et al., 2012). The standardized ginseng extract G115 (100 *µ*g/ml) was also found to increase the P2X4 dye uptake response (Fig. 1B). We investigated the effect of the individual purified ginsenoside chemicals on P2X4-mediated YOPRO-1 dye uptake responses in HEK-293 cells. To do this, we coinjected 10 *µ*M of the following ginsenosides—CK, Rd, Rb1, Rh2, or the aglycone PPD—together with ATP. Similar to the reported potentiation of P2X7 (Helliwell et al., 2015), the ginsenosides also potentiated P2X4 responses (Fig. 1, C and D). The data suggest that Rd, CK, and Rb1 can potentiate P2X4 (*P* < 0.05), whereas Rh2 and PPD had no effect on ATP-induced P2X4 pore responses in HEK-293 cells.

**Fig. 2.**
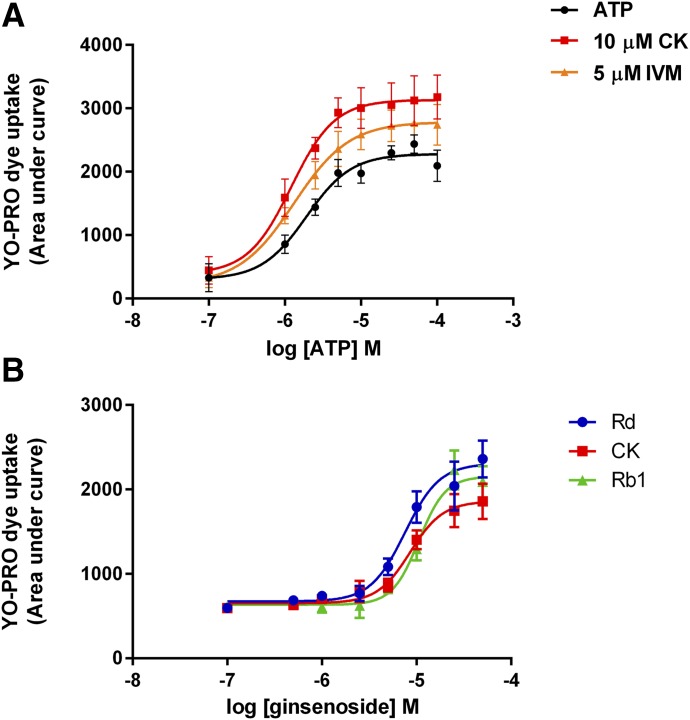
Ginsenosides enhance ATP responses at recombinant hP2X4. (A) Various concentrations of ATP (100 nM to 100 *µ*M) were used to activate hP2X4, and YOPRO-1 uptake over 200 seconds was measured on a Flexstation 3. Concentration response to ATP is shown in black, ATP in the presence of 10 *µ*M CK is shown in red, and ATP in the presence of ivermectin (IVM; 5 *µ*M) is shown in orange. Data are taken from three independent experiments, and error bars represent S.E.M. Transformed data were curve fit using nonlinear regression, and EC_50_ values were 1.92 *µ*M ATP, 1.22 *µ*M for ATP + CK, and 1.3 *µ*M for ATP + IVM. (B) Concentration response relationship for select ginsenosides on YOPRO-1 dye uptake to 5 *µ*M ATP in HEK-hP2X4 cells. Ginsenosides were tested over the range 0.1–50 *µ*M. EC_50_ values were 7.5 *µ*M Rd, 8.5 *µ*M CK, and 10.5 *µ*M Rb1. Data were taken from three independent experiments.

We investigated whether ginsenoside CK could shift the concentration–response curve to ATP and alter the sensitivity of the P2X4 receptor to the agonist. We previously reported that ginsenoside CK reduced the EC_50_ for ATP at P2X7 in addition to increasing the maximum response elicited by ATP (Helliwell et al., 2015). Using the YOPRO-1 uptake assay, 10 *µ*M CK could increase the maximum response induced by ATP, similar to the effect of the positive modulator ivermectin (5 *µ*M) (Fig. 2A). The EC_50_ value for ATP was 1.92 *µ*M (95% confidence interval 1.03 to 3.56 *μ*M) compared with 1.22 *µ*M (confidence interval 0.49 to 2.99 *μ*M) in the presence of CK, suggesting that CK does not dramatically affect sensitivity to agonist. We also determined the EC_50_ value for each ginsenoside on potentiation of hP2X4 responses (Fig. 2B), which were Rd 7.5 *µ*M (confidence interval 5.74–9.91 *μ*M), CK 8.5 *µ*M (confidence interval 5.88–12.4 *μ*M), and Rb1 10.5 *µ*M (confidence interval 8.46–13.23 *μ*M).

**Fig. 3.**
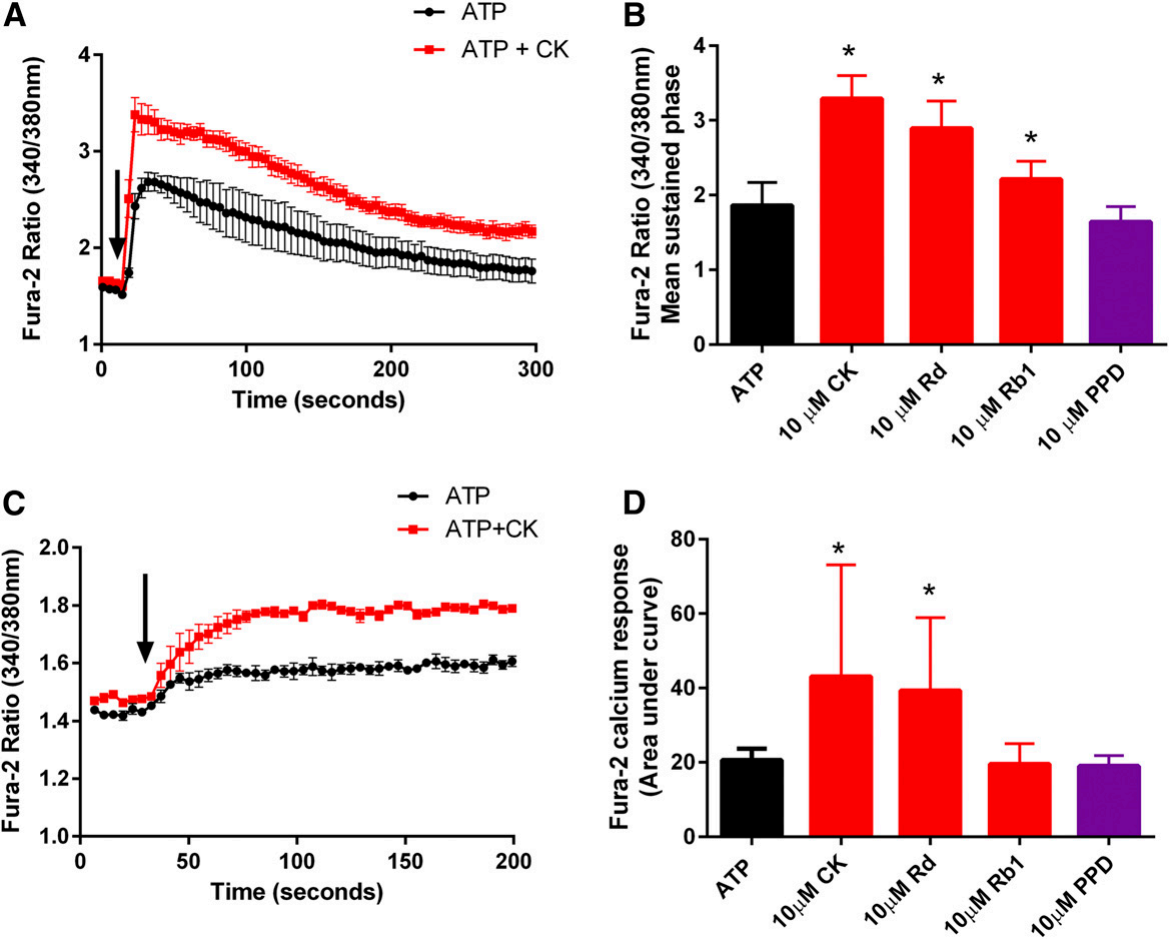
Ginsenosides enhance Ca^2+^ influx responses in cells expressing hP2X4. Intracellular Ca^2+^ responses were measured in (A) Fura-2 acetoxymethyl ester–loaded HEK-hP2X4 cells or (C) Fura-2 acetoxymethyl ester–loaded 1321N1-hP2X4 cells. Baseline values were recorded for 15–20 seconds, and then ATP was automatically injected at 30 seconds (arrow indicates agonist addition). Representative ATP responses are shown in black; ATP plus CK (10 *μ*M) are shown in red. Error bars are S.D. (B) Ca^2+^ responses in HEK-hP2X4 were calculated as average Fura-2 ratio over the final 150 seconds. CK and Rd (10 *µ*M) significantly increase hP2X4-mediated Ca^2+^ influx. *P < 0.05, one-way ANOVA with Dunnett’s multiple comparison post hoc test. (D) Ca^2+^ responses in 1321N1-hP2X4 cells were measured as area under the curve of zero baseline; error bars are S.D.; data are from four independent experiments.

**Fig. 4.**
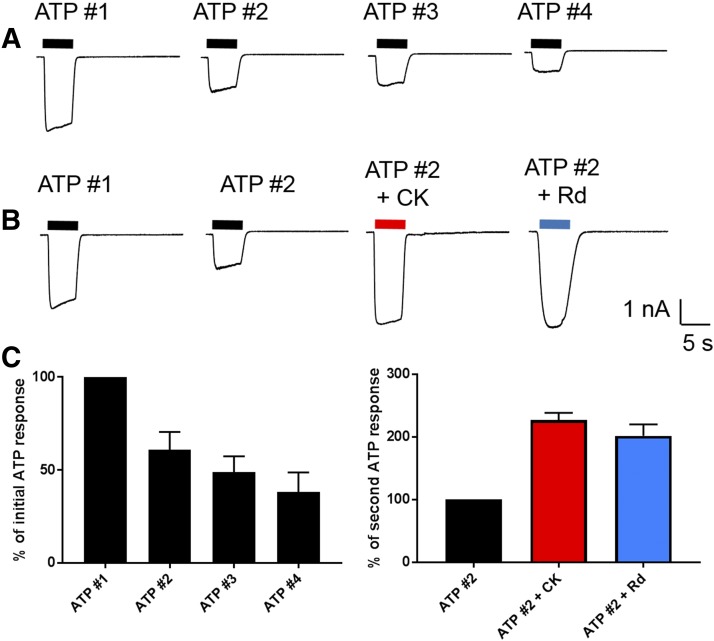
Patch clamp analysis confirms CK, and Rd enhances ATP-induced currents. HEK-hP2X4 cells were voltage clamped at −60 mV, and (A) ATP (5 *µ*M) was rapidly applied for 5 seconds (pulse denoted by black bars), followed by a 45-second washout with standard extracellular solution. In control cells, further applications of ATP were applied every 1 minute for a total of four applications. Human P2X4 responses were observed to display consistent rundown. (B) In ginsenoside-treated cells, an initial control application of ATP was administered and then the second application was ATP plus CK or ATP plus Rd (both at 10 *µ*M). (C) Normalized current amplitudes (pA/pF) showing ATP rundown over the first four applications. Potentiation of ATP responses by CK (red) and Rd (blue) expressed as percentage of second ATP response.

Our next step was to investigate the effects of ginsenosides on P2X4-mediated calcium responses using the HEK-hP2X4 stable cell line, as this is more physiologically relevant. Fura-2 acetoxymethyl ester–loaded HEK-hP2X4 cells were challenged with ATP (1 *µ*M) in the absence or presence of 10 *µ*M CK, and calcium responses were measured for 300 seconds (Fig. 3A). Endogenous P2Y receptors are known to contribute to the ATP-induced calcium responses in HEK-293 cells; however, a separate set of experiments confirmed that the ginsenosides did not potentiate ADP- or UTP-mediated calcium responses (Supplemental Fig. 1). Both CK and Rd caused an increase in the sustained calcium response mediated by hP2X4 in HEK-293 cells (Fig. 3B) (*P* < 0.05). We also generated a stable 1321N1 astrocytoma cell line expressing hP2X4 and confirmed that the ginsenosides CK and Rd potentiated ATP-induced calcium responses in this second cell type (Fig. 3, C and D). Finally, to confirm ginsenoside potentiation of hP2X4 channel responses, we used whole-cell patch clamp recordings in HEK-hP2X4 cells. As others have shown, multiple additions of ATP with a washout period in between cause a rundown effect on responses (Fig. 4A), in which the second response was 73% ± 5.8% of the initial response (*n* = 7 cells). Figure 4B demonstrates the effect of coadministering ATP in the presence of CK or Rd as the second application, showing that the ginsenosides increase the ATP responses. Figure 4C summarizes the patch clamp data as normalized data (percentage of second ATP application) (*n* = 7–10 cells).

**Fig. 5.**
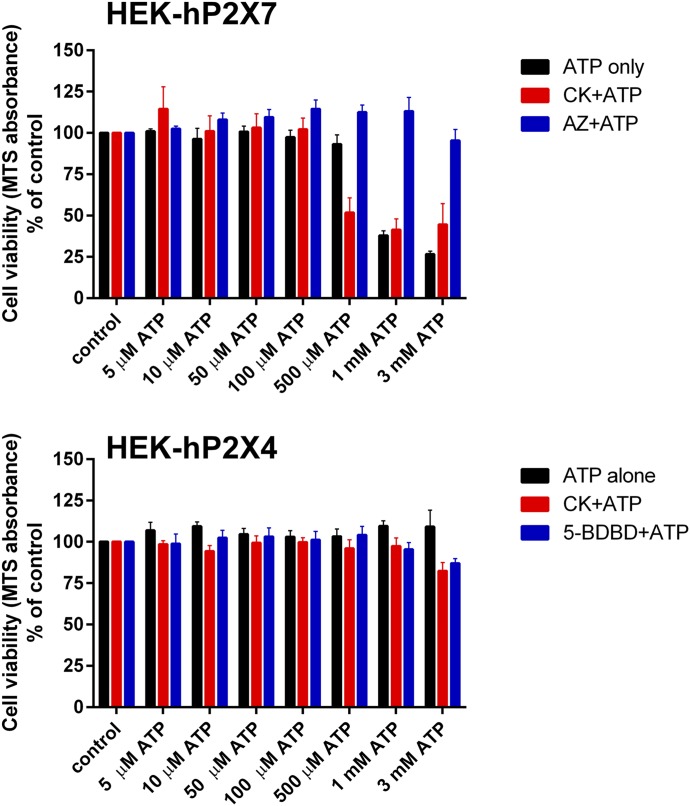
Ginsenoside CK does not enhance cell death in HEK-hP2X4 cells. HEK-293 cells stably expressing hP2X4 or hP2X7 (50,000 cells/well) were plated into 96-well plates for 24 hours prior to stimulation with ATP. Cells were pretreated with antagonists AZ10606120 (AZ) or 5-BDBD for 10 minutes before ATP was added. Cells were exposed to the indicated concentrations of ATP or ATP + CK (10 *μ*M) for 24 hours, and viability was measured using MTS absorbance. MTS was added for the final 4 hours of incubation, and absorbance read using a Flexstation 3 reader. Data were normalized to cells treated with vehicle control and are expressed as percentage of control. Data are from four independent experiments.

By measuring individual P2X receptors heterologously expressed in HEK-293 and 1321N1 cells using a variety of experimental techniques, we have demonstrated that selected ginsenosides, namely CK and Rd, can potentiate P2X4 responses. In our previous study, we investigated the downstream consequences of potentiating P2X7 receptors with the ginsenoside CK by determining its effect on ATP-induced cell death in macrophages (Helliwell et al., 2015). Therefore, we investigated whether P2X4 receptors play any role in the induction of cell death and whether ginsenosides could enhance this. We performed cell viability experiments using cell viability MTS absorbance assays in HEK-293 cells stably expressing either hP2X4 or hP2X7. In HEK-hP2X7 there was a reduction in cell viability with increasing concentrations of ATP with concentrations greater than 1 mM causing cell death (Fig. 5). The selective P2X7 antagonist AZ10606120 (10 *µ*M) could effectively block the cell death induced by these high concentrations of ATP (Fig. 5). The presence of CK enhanced the lethal effect of 500 *µ*M ATP on P2X7 in these experiments (Fig. 5). Conversely, in HEK-hP2X4 cells, there was no meaningful reduction in cell viability with increasing concentrations of ATP (Fig. 5); cells treated with 3 mM ATP were fully viable (104% of control). Viability was reduced to 86% of control when 5-BDBD was introduced in combination with high concentrations of ATP (1 and 3 mM) and viability was reduced to 76% of control when CK was introduced with 3 mM ATP (Fig. 5, *P* < 0.05).

**Fig. 6.**
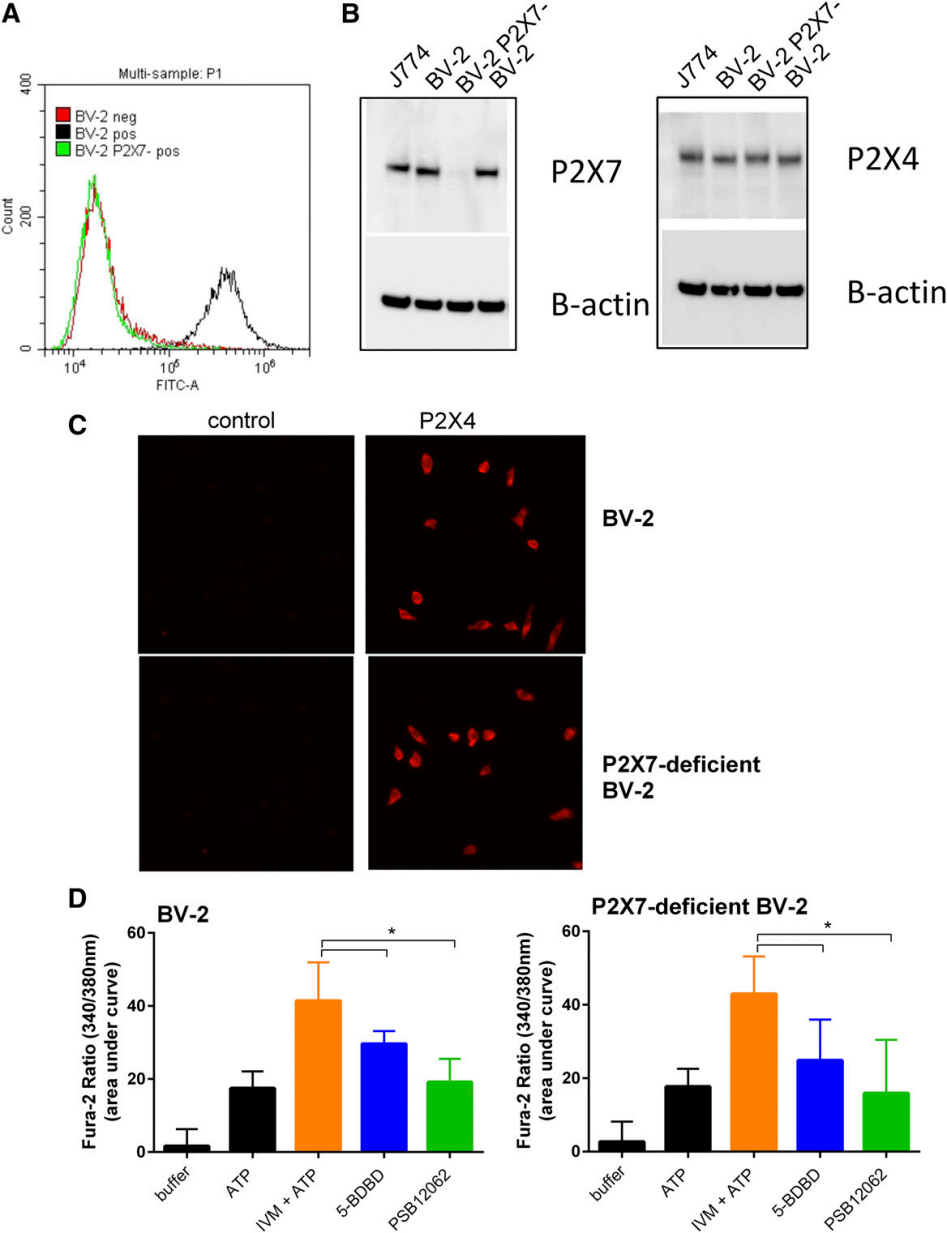
P2X4 responses in gene-edited BV-2 cells to eliminate P2X7 receptors. BV-2 cells deficient for P2X7 were generated using CRISPR/Cas9 editing. (A) Flow cytometry data indicating parental BV-2 cells express P2X7 (black histogram), whereas P2X7-deficient BV-2 cells show no P2X7-positive stain (green histogram) using Hano43 antibody labeling. Red histogram indicates negative control, cells labeled with secondary antibody only. (B) Western blots showing no anti-P2X7 reactive protein band in P2X7-deficient BV-2 cells compared with parental BV-2 and J774 macrophages. Equal amounts of protein (25 *μ*g) were loaded per lane as indicated by the *β*-actin loading control on stripped and reprobed blots. P2X4 protein levels were unchanged following knockout of P2X7. (C) Staining for P2X4 receptors was performed using a fix/perm method with cells grown on 13-mm glass coverslips. Rabbit anti-P2X4 (1:200) was used to label all P2X4 receptors in BV-2 parental and P2X7-deficient BV-2 cells. Images are representative of two independent experiments. (D) Intracellular calcium measurements in Fura-2 acetoxymethyl ester–loaded BV-2 and P2X7-deficient BV-2 show identical ATP (25 *µ*M) responses. ATP + ivermectin (3 *µ*M) responses were reduced by pretreatment of cells with 5-BDBD (10 *µ*M) or PSB-12062 (1 *µ*M). Error bars are S.D. and *P < 0.05, one-way ANOVA with Dunnett’s multiple comparison post hoc test.

**Fig. 7.**
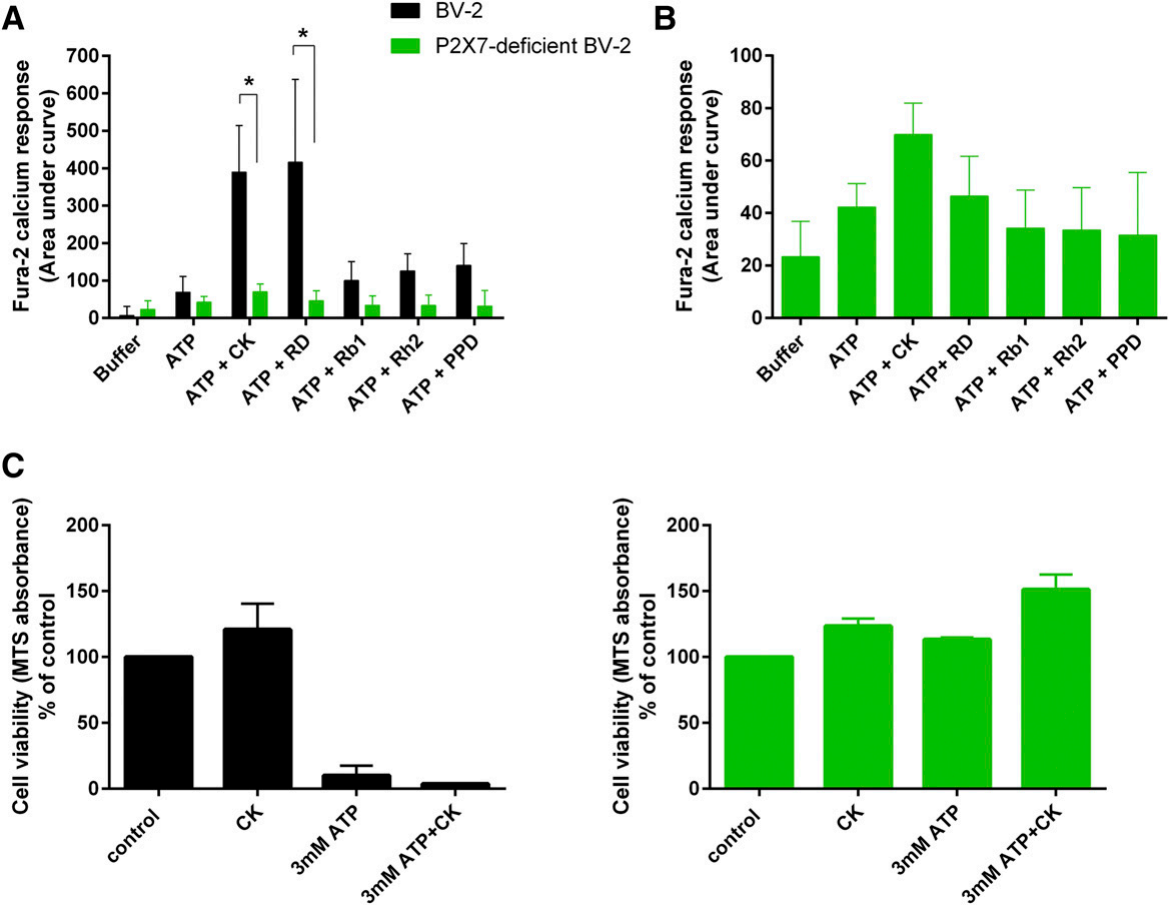
Ginsenosides show little potentiation of ATP-induced responses in BV-2 microglia in the absence of P2X7. (A and B) Intracellular calcium measurements were performed using a no-wash Fura-2-QBT assay on parental BV-2 cells and P2X7-deficient BV-2 microglia cells. ATP (200 *μ*M) was injected in the absence or presence of various ginsenosides (all 10 *μ*M). Calcium responses were calculated as area under curve using Softmax Pro software. Significant potentiation was only evident in cells expressing P2X7. Data are from three independent experiments, error bars represent S.D., and *indicates *P* < 0.05 using two-way ANOVA with Bonferroni’s multiple comparison post hoc test. (C) Cell viability was assessed using MTS reagent. BV-2 cells were plated for 24 hours prior to stimulation with ATP in the absence or presence of ginsenoside CK (10 *μ*M). Cells were treated for 24 hours, and MTS was added for the final 1 hour. Data are represented as mean percentage of control values in which control was vehicle-treated cells. Data are from three independent experiments, error bars represent S.D., and *indicates *P* < 0.05 using one-way ANOVA with Dunnett’s multiple comparison post hoc test.

To investigate the contribution of ginsenoside potentiation of P2X4 responses versus ginsenoside potentiation of P2X7 responses in immune cells, we used the mouse microglia BV-2 cell line. These cells are known to express both P2X4 and P2X7 receptors (Gilbert et al., 2016; Asatryan et al., 2018). We generated a P2X7-deficient BV-2 cell line using CRISPR/Cas9 gene-editing technology directed against exon 2 of mouse P2X7. Single-cell clones of P2X7-deficient BV-2 cells were generated and characterized using flow cytometry and Western blotting to detect surface and total P2X7 protein, respectively (Fig. 6, A and B). P2X4 protein expression was not affected in BV-2 cells lacking P2X7 (Fig. 6B), and the distribution of P2X4 appeared similar in BV-2 and P2X7-deficient BV-2 cells (Fig. 6C). ATP (25 *µ*M)-induced calcium responses were similar in the BV-2 and P2X7-deficient BV-2 cells and displayed P2X4-like pharmacological properties such as potentiation by ivermectin and reduction in responses by two P2X4 antagonists 5-BDBD and PSB-12062 (Fig. 6D, *P* < 0.05). In the parental BV-2 cells, both ginsenosides CK and Rd gave a statistically significant potentiation of the ATP-induced calcium response by 5.6-fold and 6.05 fold, respectively (Fig. 7A, *P* < 0.05 by two-way ANOVA); however, this effect was dramatically reduced in cells lacking P2X7 receptors to 1.6-fold (CK) and 1.1-fold (Rd) potentiation (Fig. 7, A and B). This suggests that the majority of the ginsenoside effect is due to their action on P2X7. We also demonstrate that parental BV-2 cells were successfully killed by 3 mM ATP, whereas P2X7-deficient BV-2 cells were protected from ATP-induced cell death (Fig. 7C). The addition of CK (10 *µ*M) together with ATP did not induce or enhance any cell death in P2X7-deficient BV-2 cells (Fig. 7C).

**Fig. 8.**
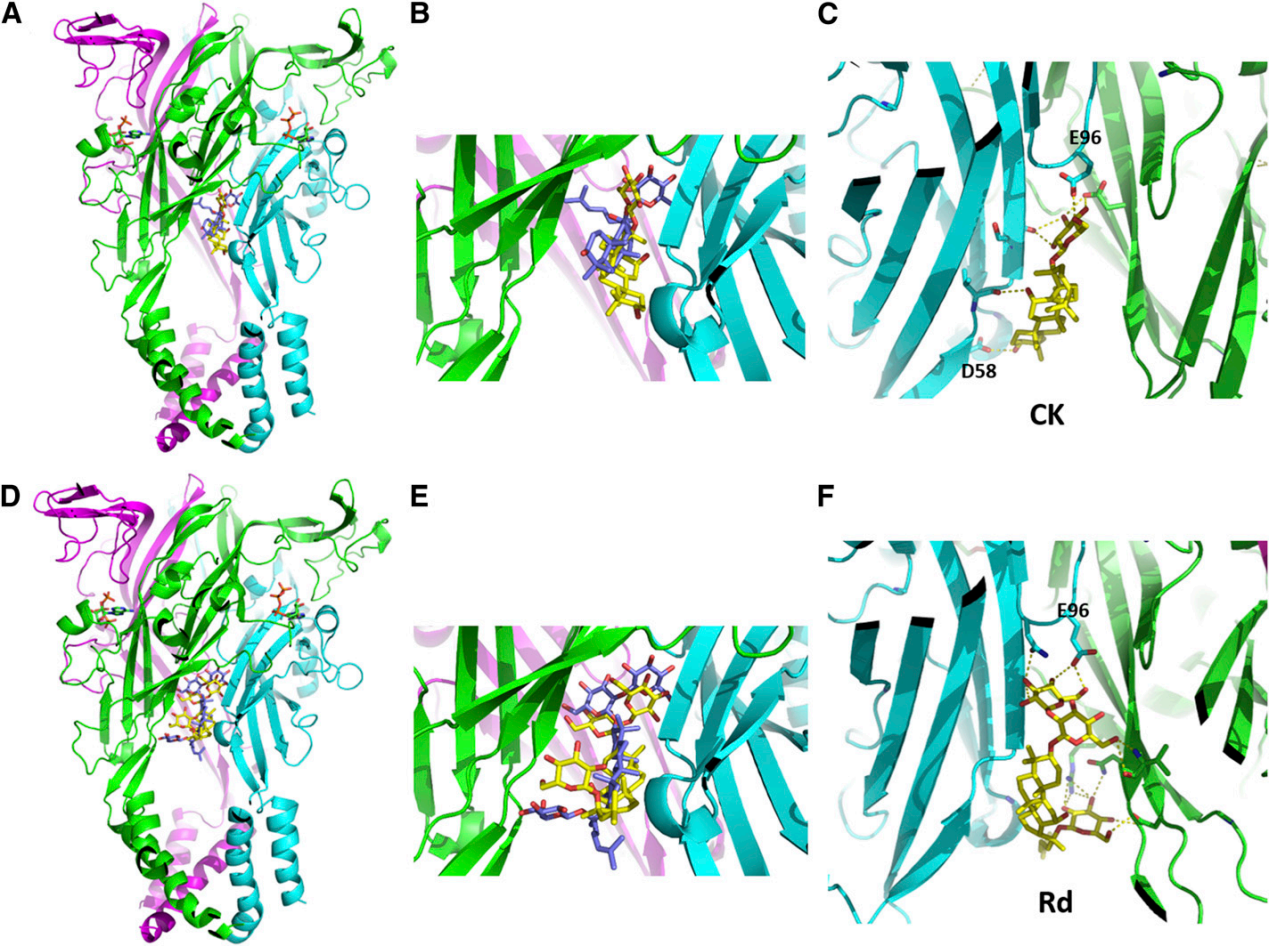
Molecular model of hP2X4 with CK and Rd docked into a predicted central vestibule binding site. (A) Representation of a homology model of trimeric hP2X4 in the open (ATP-bound) state with ginsenoside CK docked in the central vestibular region. Each subunit chain is differentially colored (green, cyan, and magenta). (B) A zoomed-in view of the binding site to compare CK pose docked to hP2X4 (yellow) to CK pose docked to hP2X7 (purple). (C) A rotated view of the CK binding site from the inside of the cavity facing outward (rotation of 180°). Polar contacts are highlighted between CK and *β*-sheets lining the lower body region. (D) Trimeric hP2X4 model with ginsenoside Rd docked into the central vestibule binding site. (E) A zoomed-in view of the binding site to compare Rd pose docked to hP2X4 (yellow) to Rd pose docked to hP2X7 (purple). (F) Same rotated view of the Rd binding site from the inside of the cavity facing outwards (rotation of 180°). Polar contacts are highlighted between Rd and *β*-sheets lining the lower body region and E96 in the internal loop region. Ligands and side chains are represented as sticks with all hydrogen atoms omitted for clarity. Images were generated in Pymol.

We have investigated putative binding sites on P2X4 and P2X7 receptors for the ginsenosides using a homology model of hP2X4 generated from the crystal structure of zfP2X4 in the ATP-bound open state (Protein Data Bank: 4DW1). The docking poses for both CK (Supplemental Material 1) and Rd (Supplemental Material 2) on hP2X4 were similar to those observed for hP2X7 (Fig. 8). There are notable differences in the length of the internal loop at the top boundary of the central vestibule, with this section being longer in hP2X4 and containing two negatively charged glutamic acid residues (-QEENS-) in comparison with neutral sequence -QGNS- in hP2X7. This additional steric repulsion of the longer internal loop in P2X4, as well as electrostatic repulsion of the diglutamate motif, may alter the binding mode of the ginsenosides compared with hP2X7. Figure 8, B and E, shows overlays of the predicted docking poses for CK and Rd on hP2X7 and hP2X4. Several interacting residues have been highlighted, including E96 in the internal loop, S62 and D58 in the *β*-2 sheet, plus D320 in the *β*-14 sheet. Ginsenoside Rd binds in an inverted orientation with respect to CK, which is similar to the docking pose observed on hP2X7 (unpublished data).

## Discussion

In this study, we have described a novel positive allosteric modulator for P2X4 and highlighted a putative binding site for positive allosteric modulators that may be conserved between P2X4 and P2X7 receptors. We have presented pharmacological data for positive modulator activity at P2X4 and have described important differences between ginsenoside action at P2X4 and P2X7.

To measure P2X4 responses, we used a YOPRO-1 dye uptake assay, which is most commonly used to measure P2X7 responses. Earlier works suggested that dye uptake responses could also be induced by other purinergic P2X receptors, including P2X4 (Khakh et al., 1999a; Virginio et al., 1999), and this was confirmed by Bernier et al. (2012) in both transfected HEK-293 cells and microglia. This might suggest that dye uptake is an intrinsic feature of P2X receptor channels. Regardless of the mechanism underlying permeation of large dye molecules, this is a potentially useful way of measuring P2X4 responses in a medium- to high-throughput system. We show that dye uptake was induced by low concentrations of ATP, was potentiated by ivermectin, and was reduced by pretreatment of the cells with 5-BDBD, all characteristics of a P2X4-mediated response. In these experiments, CK appeared to be more effective at enhancing ATP-induced responses than ivermectin (Figs. 1 and 2).

Testing the protopanaxadiol ginsenosides on P2X4 in YOPRO-1, calcium measurements and patch clamp recordings revealed some differences to P2X7; the most striking difference was that Rd was equivalent to CK in the level of potentiation it induced on P2X4, whereas CK is more effective at P2X7. Overall, the magnitude of potentiation observed for P2X4 was much lower than on P2X7; ginsenosides typically increased P2X4 responses by ∼twofold, whereas this reached >20-fold in P2X7 responses by patch clamp analysis (Helliwell et al., 2015). The similar PAM action on P2X4 and P2X7 prompted an investigation into whether the predicted binding site for ginsenosides would be the same. There is a high degree of similarity between these channels, and we used the zfP2X4 crystal structure to predict the ATP-bound open states of hP2X4 and hP2X7. Differences in the binding poses were observed and in amino acid residues predicted to interact with bound ginsenosides, the roles of which will be investigated in a more detailed study. It is notable that the predicted ginsenoside site does not overlap with the ivermectin binding site on P2X4, which is thought to be located within the transmembrane domains (Jelínková et al., 2006; Silberberg et al., 2007; Popova et al., 2013), suggesting that two distinct positive modulator binding sites exist for P2X4.

In this study, we have paid particular attention to the cytotoxic action of ATP. It is well known that sustained activation of P2X7 leads to cell death (Orioli et al., 2017), and we have shown that ginsenosides can enhance this (Helliwell et al., 2015). The role of other purinergic receptors in cell death is not so clear, and therefore we investigated the involvement of P2X4 in this process. We found that sustained activation of hP2X4 overexpressed in HEK-293 cells did not cause cell death (Fig. 5), and furthermore, high concentrations of ATP were not cytotoxic in P2X7-deficicent BV-2 microglial cells (Fig. 7), suggesting that activation of P2X4 does not cause cell death. In HEK-hP2X7 cells, CK was able to enhance cell death to nonlethal concentrations of ATP (500 *µ*M), similar to J774 cells (Helliwell et al., 2015). This is likely due to the increased receptor sensitivity to ATP in the presence of CK, allowing cells to overcome a threshold level of signaling for the initiation of apoptosis. CK was not as effective at enhancing cell death in response to 1 or 3 mM ATP; however, this may be due to maximum cell death being induced by full activation of P2X7.

Positive allosteric modulators for P2X4 are thought to be useful in treatment of alcohol-use disorders due to a competitive effect on channel activity (Asatryan et al., 2010; Yardley et al., 2012). Ethanol has an inhibitory effect on P2X4 responses and prevents the action of ATP, which in turn affects the modulation of neurotransmission in various regions of the brain (Franklin et al., 2014). The binding of ivermectin to P2X4 is postulated to counteract the binding of ethanol, thus preventing its inhibitory effect on channel activation. It would be interesting to determine whether positive allosteric modulators that bind to other regions of the channel will have a similar effect on elimination of ethanol effects on P2X4. In addition to alcohol-use disorders, P2X4 has been implicated in the regulation of dopamine-dependent behaviors (Khoja et al., 2016), suggesting that modulation of P2X4 in the CNS may also be beneficial in dopaminergic disorders such as Parkinson’s disease. Interestingly, *P. ginseng* extract is reported to have some beneficial effects in animal models of Parkinson’s disease (Van Kampen et al., 2014). However, due to their chemical properties, it is unclear how easily the ginsenosides cross the intact blood-brain barrier. A recent study suggests ginsenosides can be detected in rat vascular endothelial cells and astrocytes following oral administration of high purity ginseng total saponins (Zhao et al., 2018). The larger ginsenosides with multiple sugar moieties are much less likely to be absorbed from the intestine into the bloodstream, but the smaller ginsenosides and metabolites such as CK are found at appreciable concentrations in plasma and liver. Peak plasma concentrations of CK are reported to be 8.35 ng/ml (low nM range) in humans following oral administration of Korean red ginseng extract (Kim, 2013), although a more recent study found higher plasma concentrations (1183.2 ± 445.1 ng/ml following a single 800 mg dose) in volunteers receiving pure Ginsenoside CK tablets (Chen et al., 2018). Furthermore, the source of the ginsenosides, whether from a natural extract, supplement, high purity chemicals, total saponins, plus the route of administration, length of dosing, and the influence of the gut microbiota are all factors that are likely to affect plasma levels. It also remains unclear whether reported CNS effects are due to peripheral effects. As these natural products gain recognition for their potential therapeutic benefits, more detailed studies are likely to be conducted in humans.

There are several processes in which P2X4 has been shown to play a role; in endothelial cells P2X4 is reported to be involved in a vasodilatory effect induced by shear stress (Yamamoto et al., 2000), and in the hippocampus P2X4 has a role in long-term potentiation (Sim et al., 2006). Enhancement of these physiologic processes may be beneficial in people suffering from hypertensive disorders or cognitive/memory deficit, and positive allosteric modulators may be a useful future strategy for manipulating P2X4 receptors. It has also been shown that activation of P2X4 can cause surfactant secretion in the lung (Miklavc et al., 2013), chemokine and prostaglandin E2 secretion from macrophages (Ulmann et al., 2010; Layhadi et al., 2018), and BDNF secretion from spinal microglia (Coull et al., 2005; Ulmann et al., 2008). It is less clear whether enhancement of such responses would be beneficial, particularly in the case of the P2X4-BDNF axis in neuropathic pain. However, we have shown that the action of CK and Rd ginsenosides on P2X4-mediated calcium responses in microglial cells is minor (Fig. 7), and this may be due to the low surface expression of P2X4 in these cells (Qureshi et al., 2007; Stokes and Surprenant, 2009). Alternatively, the ginsenosides may be less active on mouse P2X4 receptors relative to human P2X4 receptors, because the BV-2 cell line is of mouse origin. It is unlikely that ginsenosides would enhance neuropathic pain because concentrations in the CNS would be much lower than plasma concentrations and below the levels reported in this study to potentiate P2X4. Peripheral P2X4 receptors are therefore more likely to be modulated by circulating ginsenosides or ginsenosides accumulated in tissues such as liver and lung.

In conclusion, we have demonstrated that ginsenosides are not selective in their potentiating action on P2X7 receptors, but have some PAM activity on P2X4 receptors most likely through a similar binding site in the large ectodomain of these trimeric channel complexes.

## Abbreviations

ANOVA: analysis of variance
BDNF: brain-derived neurotrophic factor
BSA: bovine serum albumin
CK: compound K
CNS: central nervous system
DMEM: Dulbecco’s modified Eagle’s medium
DMSO: dimethylsulfoxide
MTS: 3-(4,5-dimethylthiazol-2-yl)-5-(3-carboxymethoxyphenyl)-2-(4-sulfophenyl)-2H-tetrazolium, inner salt
PAM: positive allosteric modulator
PBS: phosphate-buffered saline
PPD: protopanaxadiol
RMSD: root-mean-square deviation
TBST: Tris-buffered saline/Tween 20
